# Maternal and Fetal Death following Group A Streptococcal Meningitis in Mid-Term Pregnancy

**DOI:** 10.1155/2014/268693

**Published:** 2014-05-04

**Authors:** Sayinthen Vivekanantham, Nadeesha Mudalige, Venothan Suri, Abderahman Kamaledeen, Penelope Law

**Affiliations:** ^1^Imperial College School of Medicine, Imperial College London, London SW7 2AZ, UK; ^2^The Devonshire Lodge Practice, 12A Abbotsbury Gardens, Eastcote, London HA5 1TG, UK; ^3^King's College London, Strand Campus, London WC2R 2LS, UK; ^4^The Hillingdon Hospital, Uxbridge, London UB8 3NN, UK

## Abstract

*Background*. Group A streptococcal (GAS) meningitis is rarely seen in the antenatal period, but it is associated with significant mortality. We present a case of a mid-trimester woman who developed fulminant meningitis following a rapid onset atypical presentation of infection with this organism. *Case*. A multiparous 23^+5^-week woman presented with a 10-day history of a non-productive cough associated with pyrexia. Within minutes of her admission she collapsed and lost consciousness; sepsis was suspected and cross-specialty care was initiated. She was managed empirically in extremis with broad-spectrum antibiotics and mannitol with 3% hypertonic saline for suspected infection and raised intracranial pressure, respectively. Despite intensivist management, a CT head revealed diffuse oedema with coning of the cerebellar tonsils. Brainstem death was certified within 19 hours of admission and fetal death ensued. Postmortem bacteriology confirmed GAS meningitis. *Conclusion*. Through raising awareness of this patient and her disease course, we hope that future policy decisions, primary care, and hospital level management will be informed accordingly for treatment of pregnant women with suspected GAS infection.

## 1. Introduction

Group A Streptococcus (GAS) is commonly observed within the community and remains a leading cause of otolaryngological and skin infections [1]. However, GAS meningitis is a rare manifestation, accounting for 2% of pyogenic meningitis in the United States [2] and The Netherlands [3], that is associated with a significant mortality, approximately 25% [3]. The high virulence of GAS is attributed to the production of pyrogenic exotoxins which induce a cytokine storm and aberrant immunological activation, leading to septic shock [4]. We present a case of a mid-term mother who died following a rapid onset atypical presentation of this organism. To our knowledge, this is the first case of fatal GAS meningitis in mid-trimester pregnancy documented in the literature. We hope this report will act as a discussion platform for recommendations and guidance made in* The Eighth Report on Confidential Enquiries into Maternal Deaths in the United Kingdom* (CMACE) too [5].

## 2. Case

A 32-year-old Pakistani woman, who was 23^+5^ weeks into her fourth pregnancy, presented to her general practitioner with a seven-day history of a nonproductive cough associated with pyrexia. Incidentally, her entire family had been unwell with similar symptoms. We are unaware of the general practitioner's examination but understand that a viral throat infection was suspected and she was advised to rest and keep good fluid intake.

Three days later she presented to accident and emergency (A&E). She complained of persistent cough in conjunction with nausea, significant malaise, and visual changes. Within four minutes of walking into A&E, the patient collapsed with loss of consciousness. Her baseline observations were HR 160, BP 103/42, temperature 36.9°C, and GCS 5/15. However, her condition rapidly deteriorated. Within 15 minutes she desaturated with bilateral poor air entry. In addition, her circulation was compromised (BP 60/40, HR 195), so aggressive fluid resuscitation was commenced with the support of noradrenaline and vasopressin. Furthermore, a neurological assessment revealed that her left pupil was fixed and dilated. According to empirical guidance, magnesium sulphate and treatment dose of low-molecular-weight heparin were given for suspected atypical presentation of eclampsia and pulmonary embolus, respectively. On examination, a pendulous abdomen was observed with a palpable uterus extending just above the umbilicus. Her cervix was long and her external ostium was closed, with no signs of per vaginal bleeding. Fetal movements were palpated and a viable fetus was confirmed on ultrasound. 12 mg intramuscular dexamethasone was also administered.

Differential diagnoses considered at this stage included maternal sepsis (bacterial meningitis, viral encephalitis, or respiratory tract infection), a thromboembolic event, intracranial hemorrhage, and eclampsia. A full septic screen was conducted, hematological investigations indicated infection (WCC 30.6 × 10^9^/mL, CRP 153 mg/L), and biochemistry revealed metabolic acidosis (pH 7.18, lactate 2 mmol/L) with deranged renal function (creatinine 102 *μ*mol/L). As a result, broad-spectrum antibiotics (2 g ceftriaxone, 500 mg metronidazole) and antiviral cover (acyclovir 900 mg, zanamivir 10 mg, and oseltamivir 75 mg) were commenced in accordance to empirical guidance from the microbiology department.

In light of her unresponsive pupil, an immediate computed tomography head scan was arranged. This showed generalized cerebral edema with cerebral tonsillar herniation; there was no focal intracranial hemorrhage. Mannitol and hypertonic (3%) saline were administered to lessen the cerebral edema. The imaging was reviewed by the on-call neurosurgical team at a tertiary center which recommended attempting to decrease the cerebral edema further. She had a normal chest X-ray and normal urinalysis, so the primary focus of sepsis was suspected to be meningeal. A lumbar puncture was contraindicated due to her raised intracranial pressure.

The patient remained sedated and ventilated under intensive care management. The regional neurology center again reviewed the images; unfortunately the damage to the brainstem, cerebellum, and frontal lobes at this stage was irreversible. A brainstem test on the mother at 19 hours after admission showed no signs of life, which was confirmed 12 hours later. At 20 hours of admission an ultrasound scan confirmed the fetal heartbeat was lost at 23^+6^ weeks. Postmortem bacteriology confirmed streptococcal Group A meningitis as the cause of death.

## 3. Discussion

GAS meningitis is rarely observed in adults and accounts for less than 0.3% of cases [6]; nevertheless, it is associated with a high level of mortality (10–30%). Obstetricians are likely to encounter streptococcal infections in the puerperium, whereby the organism that colonizes the lower genital tract often triggers ascending infections. In the context of reduced immunological barriers and retained products of conception, this can cause widespread sepsis. Whilst a recent account of antenatal GAS meningitis has been reported, this was not fulminant and was in the latter stages of pregnancy [7]. This report serves as reminder to obstetricians of the atypical manifestation of GAS meningitis and its high level of virulence. We will discuss certain aspects of her care and the ethical issues that come to prominence.

**Figure 1:**
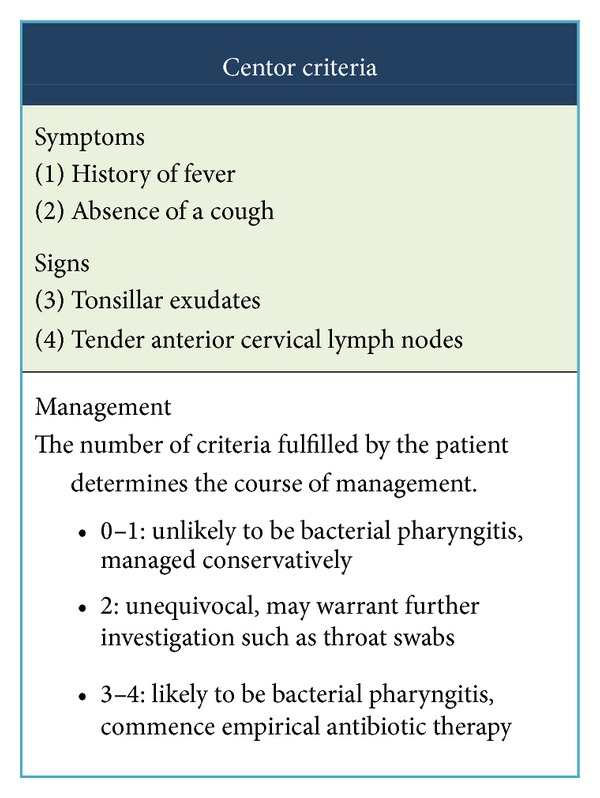
Centor criteria.

Our patient presented to her general practitioner three days before her admission and was assessed using the Centor criteria (Figure 1) [8, 9], a crude predictor of GAS pathogenesis in pharyngitis. In the UK, it is not recommended that throat swabs and rapid antigen tests should be performed routinely, as there is validity to the argument that these tests would not differentiate between infection and carriage [9]. Nevertheless diagnostic investigations may be merited in the higher-risk antenatal population. In a review of GAS meningitis by Chow and Muder [10], it is highlighted that this organism rarely infects the meninges before forming a primary focus of infection elsewhere. It is likely in our patient that GAS spreads contiguously from the oropharynx through the Eustachian tube to the middle ear and subsequently via the internal acoustic meatus to the meninges. In light of this report, we suggest that clinicians have a lower threshold for the use of antibiotics in throat infections during pregnancy. This is supported by a study into maternal deaths in The Netherlands between 1983 and 2007, in which nine out of 15 (60%) cases with fatal meningitis had an otolaryngological infection at presentation or in the preceding days [11].

There are a number of different assessment tools used to assess the probability of GAS infection in children [12, 13] and in adults [9, 14–16]. Although new assessment tools have been suggested [17–20], in the UK the Centor criteria [9] are widely used in both primary and secondary healthcare settings. The appropriate management to follow depends on the presence of different risk factors that have been described by Snow et al. [21]. However, there have been ongoing discussions about the appropriateness of the Centor criteria in nonimmunocompromised adults [1, 22] and for use in a low prevalence setting [8]. From our assessment at hospital, given that there was no documented examination of lymph nodes, our patient only fulfilled a possible maximum of two points on the Centor Criteria (history of fever was present). This suggests that the risk of bacterial pharyngitis is low enough to not warrant antibiotics without further investigation in a nonimmunocompromised patient. Hence, we suggest that the Centor criteria should be used with caution during the relatively immunocompromised state of pregnancy. Perhaps a lower threshold for use of antibiotics for throat infection should be incorporated into guidelines for those who are immunocompromised, especially in low threshold settings where the Centor criteria hold less value [8]. We would also like to highlight the role of the GP in safety netting of these patients; they should also be made aware of the cardinal symptoms of meningeal infections and the need for prompt medical attention if these symptoms develop.

On admission to hospital, the patient was moribund and decompensated further within minutes. The CMACE report in 2011 highlights that sepsis is now the leading cause of direct maternal death, having increased from 0.85 to 1.13 per 100,000 maternities, largely from community acquired GAS. The report warns that* “…staff need to be aware that women … may appear deceptively well before suddenly collapsing, often with little or no warning.”* Our presentation supports the warning in CMACE report that both BP and HR can be maintained until very late stages in sepsis [5] and hence should not be used as sole parameters for assessment of the febrile gravid patient. Furthermore, once established, sepsis may be fulminating with rapid deterioration into septic shock, disseminated intravascular coagulation, and multiorgan failure [5]. Several measures are important in the management of the patient that can influence the odds of survival.

In certain situations, delivery helps to reduce the degree of cardiovascular compromise during maternal sepsis. However in this case, there was no sign of fetal infection, no significant inferior vena cava compression nor significant diaphragmatic splinting. Furthermore, the patient was too unstable to undergo delivery by caesarian section and so delivery was not indicated. During the resuscitation, sepsis care pathways were initiated and antibiotics were infused. Furthermore, effective multidisciplinary cross-communication was observed between the obstetric, emergency, intensivist, neurosurgical, medical and midwifery teams, which is consistent with the latest Royal College of Obstetricians and Gynaecologists guidelines (UK). The CMACE report also supports the early use of clindamycin over penicillin due to inhibition of exotoxin production by GAS, within the “golden hour” following maternal collapse [5].

This case also emphasises the typical demography of sepsis in pregnancy. A significant number of maternal deaths due to sepsis during 2006–2008 were from an ethnic minority, had young children, and presented during the winter months [5]. Our patient also demonstrated a moderate language barrier, so we emphasize the importance of professional interpreting services in the hospital setting, which is consistent with the recommendations made in the last CEMACE report.

## 4. Conclusion

This case serves as a reminder that obstetric patients are particularly vulnerable to fulminant sepsis of any nature, but in particular GAS. The role of the general practitioner remains central to the early diagnosis and treatment of these patients, although current guidelines for suspected GAS have not been appropriate in our patient, which has ultimately led to her demise. We suggest that lowering the threshold for intervention on the Centor criteria for obstetric patients may be appropriate. We encourage further research on presentations of obstetric patients and other immunocompromised individuals with GAS to assess whether an addition to the criteria for such patients would be beneficial from a public health standpoint.
